# Daily and seasonal human mobility modulates temperature exposure in European cities

**DOI:** 10.1371/journal.pone.0330912

**Published:** 2025-09-03

**Authors:** Guo-Shiuan Lin, Maider Llaguno-Munitxa, Gabriele Manoli

**Affiliations:** 1 Laboratory of Urban and Environmental Systems, School of Architecture, Civil and Environmental Engineering, École Polytechnique Fédérale de Lausanne (EPFL), Lausanne, Switzerland; 2 Louvain Research Institute of Landscape, Architecture, Built Environment, Université Catholique de Louvain (UCLouvain), Ottignies-Louvain-la-Neuve, Belgium; The Chinese University of Hong Kong, HONG KONG

## Abstract

Extreme temperatures pose a serious threat to human health, especially in urban areas where the majority of the world population is living. Temperature-related risks are exacerbated by urban-induced warming but existing exposure assessments rely on a static residential population, thus overlooking space-time changes in population density and their covariation with urban temperatures. Here we combine 1-km monthly daytime and nighttime population estimates for 80 European cities with existing high-resolution urban climate simulations to quantify the impact of daily and seasonal mobility on residents’ exposure to heat and cold. Using city-specific exposure-response curves and the respective minimum mortality temperatures as thresholds to define hazardous conditions we calculated that, on daily timescales, commuting towards city centers causes a 7.8% average increase (IQR:1.0-12.9%) in summer heat exposure but, during winter, it provides a slight protective effect against cold. On seasonal timescales, changes in total population are out of phase with the temperature cycle in most European cities, leading to a lower exposure to heat, with the exception of touristic destinations where exposure increases, on average, by 0.9% during the warmest months. These results highlight the key role of human mobility for heat risk assessment and adaptation and they reveal the existence of general exposure trends that hold across diverse cities and climates.

## Introduction

Exposure to heat and cold has negative impacts on human health, thermal well-being, and economic productivity. High ambient temperature is associated with higher mortality rates from cardiovascular and other diseases [1], negative effects on mental health [2], and lower work performance [3] while low ambient temperature is associated with excessive mortality from cardiovascular, cerebrovascular, and respiratory diseases [4,5]. With more than 50% of the global population now living in cities [6] and a projected increase to 70% by 2050 [7], urban areas have become hotspots of temperature-related risks [8–10]. Human exposure to high outdoor temperatures is rapidly increasing due to global climate change, which is already causing an increase in temperature-related morbidity and mortality [11,12]. Urbanization, by modifying the surface energy balance and generating the so-called urban heat island (UHI) effect [13–15], can further amplify the health impacts of extreme heat as both the UHI intensity and the density of urban dwellers generally increase towards city centers [16,17]. This co-occurrence of high temperatures and high population density has been observed to increase heat exposure (e.g., by 1 ${\;\;}^{\circ }C$ in the UK [18]) and recent evidence suggests that UHIs can cause a 45% median increase in mortality risk during summer [19]. Such urban climate-related risks are expected to rise in the future as - in addition to regional warming - cities around the world will also experience an urbanization-induced temperature increase that can exacerbate locally the impact of global climate change [20,21].

Quantifying the level and spatial distribution of hazardous temperature exposure in cities can help identify heat-risk hotspots and develop targeted mitigation/adaptation strategies. As such, heat risk assessments are being conducted in cities worldwide [22–25]. However, while many studies incorporated long-term (i.e., 10 to 100 years) population growth and urban warming [26–30], how short-term (i.e., daily and seasonal) population dynamics influence heat risk has received much less attention. Existing heat risk assessments generally use static population data to estimate temperature exposure, thus assuming that people stay at their registered home address throughout the entire study period (e.g., [19,31,32]). However, humans routinely travel to different cities and neighborhoods for various purposes, which temporarily increases/decreases urban population density and reshapes its spatial distribution. On seasonal timescales, the presence of workers, students, and tourists can cause significant fluctuations in populations [33] while, on a daily basis, people commute to city centers from neighboring areas to work, study, or access a variety of urban services and amenities. For example, in Europe 62% of the population takes at least one touristic trip per year (data code: tour_dem_totot [34]) and about 97% of the employed population mainly worked outside their homes before COVID-19 (data code: lfso_19plwk25, [34]). As a consequence, daytime population in city centers is significantly higher than their respective nighttime population, with estimates of daytime/nighttime ratios ranging from 1.49 (for Chicago, USA [35]) to 1.9 (for the 31 largest cities in Europe [17]). These changes in urban population are heterogeneous both in time and space as activity hotspots such as central business districts, airports, universities, and sport stadiums attract more people than other areas during specific periods or times of the day [17,36].

In light of these mobility patterns, it is clear that census data represents at best the nighttime population distribution, and cannot reflect the actual exposure of people to urban daytime conditions. This issue is well known in the context of air pollution where granular measurements and individual-level exposure assessments are increasingly considered (e.g., [37–40]), but the impact of mobility on temperature-related risks remains largely unexplored. The study by Hu et al. [35] for the metropolitan area of Chicago was among the first attempts to investigate the concurrent variations of daytime population and temperature within a city, followed by the work by Yang et al. [41] who quantified that exposure to extreme heat is intensified by about 1.9 °C when considering commute-adjusted population distributions in the 16 major metropolitan areas of the US. More recently, Yin et al. [36] have proposed a dynamic thermal exposure index (DTEx) to identify high-exposure locations in the Athens-Clarke County (Georgia, US), while Huang et al. [42] investigated mobility patterns in 20 US metropolitan areas and identified the emergence of “heat traps”, where population residing in high-heat neighborhoods visit other high-heat areas.

The above-mentioned research showed that mobility can greatly alter population distribution and thus heat exposure. Despite progress on this front, several issues remain unresolved. First, existing research has predominantly focused on daily timescales, thus neglecting the potential impact of seasonal mobility on heat/cold exposure. Recent evidence suggests that the negative impacts of UHIs can be counterbalanced by a protective effect in winter, when UHIs can shield against extreme cold events [19,43]. Yet, the potential modulating effect of population dynamics in different cities and periods of the year is largely unknown. Second, the focus of existing studies has remained on a few metropolitan areas in the US at a coarse resolution (i.e., 5 km in Hu et al. [35] and Yang et al. [41]), thus limiting the generalizability of the results to other geographic contexts. Europe, a region facing increasing heat risks [12,44,45], still mainly rely on static residential population for heat exposure studies (e.g., [19,45]). Finally, existing dynamic assessments solely consider temperature and population distributions without considering city-specific vulnerability to temperature changes.

In this study, we aim to (1) assess how and to what extent population dynamics influence the exposure of city dwellers to urban temperatures as compared to traditional, static, assessments and (2) identify differences and/or regularities in the dynamics of temperature exposure and risks across diverse European cities. We analyze how daily and seasonal human mobility modulates heat/cold exposure and risk in 80 European cities by combining monthly daytime and nighttime population estimates at 1-km resolution [17] with urban temperature simulations and city-specific temperature exposure-response functions (ERF) [8,19]. ERFs encapsulate people’s ability to cope with heat and cold (i.e., their vulnerability [19]), thus allowing to convert mobility-induced changes in exposure to preliminary estimates of city-specific risk (see Materials and Methods). Here we define heat/cold exposure as the amount of population exposed to climatic conditions deviating from the optimal temperature of each city (as derived from the city-specific ERFs) while heat/cold risk is defined as the population-weighted relative mortality risk (also derived from the ERFs, see Materials and methods). We start by categorizing aggregated daily and seasonal mobility flows to unravel their associations with urban temperatures and city size. Subsequently, we elucidate the correlation between intra-urban population and temperature distribution and their dependence on proximity to the city center. Finally, we estimate the impact of daily and seasonal mobility on temperature-related exposure and risks. Our findings provide the first dynamic quantification of urban population exposure in Europe and uncover broad regularities in the mobility-exposure relationships observed in different cities and climates. We argue that a high-resolution quantification of mobility-adjusted exposure can better reveal the actual spatial and temporal changes in temperature-related risks, identify high-risk regions and periods, and guide heat mitigation strategies that consider human behavior. Note, however, that the focus here remains on typical commuting/traveling patterns (defined as monthly averaged population estimates) and we disregard how people may potentially adjust their mobility in response to extreme temperatures [46]. This is considered beyond the scope of this study but it represents an exciting subject for future research.

## Materials and methods

### Urban climate simulations

Air temperature estimates are obtained from existing urban climate simulations run with UrbClim [47,48], an urban boundary layer climate model consisting of a land surface scheme coupled with a three-dimensional atmospheric boundary layer module. The latter is tied to hourly synoptic meteorology from European Centre for Medium-Range Weather Forecasts Reanalysis v5 (ERA5) [49] through lateral and top boundary conditions. The urban terrain in the land surface scheme is represented as an impermeable slab with parameters for albedo, emissivity, and aerodynamic and thermal roughness length, and accounting for anthropogenic heat fluxes. UrbClim has been extensively validated with observation across Europe and showed fairly good accuracy [47,48]. The simulation outputs for 100 European cities at hourly and 100-m resolution were made available on the Copernicus Climate Change Service [50] (data retrieved on June 2023). Here, we employ daily 2-m air temperature simulations from 2010 to 2012 for the 80 European cities where the corresponding ERFs are available from Huang et al. [19]. The time period includes relatively usual years for European temperatures without exceptional heat/cold extremes and are consistent with the time of the population dataset (see next section). Daily average temperatures are re-gridded to 1-km resolution to be at the same resolution as the population data. The sea-land mask from the same dataset is used to exclude water surfaces when calculating the urban domain mean temperature and constructing cities’ radial profiles (see Materials and Methods). The main advantage of this dataset is its higher spatial resolution compared to previous studies (e.g., 5 km in Hu et al. [35] and Yang et al. [41]) which improves the accuracy of the exposure calculation, especially for European cities as they are typically smaller than the US cities analyzed in the aforementioned studies.

### Monthly daytime and nighttime population estimates

In this study we use the spatial raster dataset of monthly daytime and nighttime population counts at 1-km resolution covering the 28 Member States of the EU derived by Batista e Silva et al. [17]. The nighttime frames assume the entire population is at their registered residence or temporary lodging (for tourists). Conversely, the daytime frames presume people to engage in their primary daytime activities, such as working, studying, etc., or possibly staying at home. As such, the difference between these daytime and nighttime frames represents the daily population variations caused by the primary commuting behavior. Monthly variations in population were estimated using country-specific school and holiday calendars, along with official statistics on monthly inbound and outbound tourists.

The daytime and nighttime population distributions were derived by Batista e Silva et al. [17] according to the following steps. First, 16 population sub-groups were distinguished for each small statistical region based on their main activities (e.g., employees, tourists, students, non-working and non-studying population). Second, land use features were mapped at 100-m resolution based on 18 specific classes (e.g., green urban areas, commercial or service facilities, university locations, airport terminals, etc.) for the allocation of the population subgroups. In addition, Point-of-Interest data and tourist accommodation room density data from online booking platforms were also used for allocating population to complement the land use map. Third, each population subgroup was aggregated within the regions to its most likely locations based on the first and second steps. Finally, the counts from all population subgroups were summed to get the total population at each grid cell. The main advantage of this dataset is its extensive spatial coverage — entire Europe at 1 km resolution — which enables the comparison of mobility behaviors across multiple cities, offering new insights beyond previous studies that typically focused on individual cities (e.g., [35,36,51]).

Despite a generally good agreement with other independent sources of daytime population, certain cities, such as Madrid, Barcelona, Valencia, Athens, and Stockholm, exhibit an unexpected pattern where the daytime population is lower than the nighttime population [17]. This anomaly is primarily attributed to the likely underestimation of daytime population [17]. Due to this potential bias, our study does not emphasize on the effects of such daily mobility patterns on temperature exposure (see Supporting Information). In addition, we should note that the dataset has a few additional limitations, such as the omission of sub-daily population dynamics, the lack of distinction between weekends and weekdays, and the lack of information on the origin and destination of individuals (i.e., origin-destination matrix). Besides, the nighttime and daytime frames for each month were derived from the reference year 2011, which corresponded to the latest round of European censuses before the study, thus disregarding any post-COVID change in mobility and behaviour. Yet, it is reasonable to assume that the main urban structure and population distribution was not altered significantly over the past years. It also does not account for the fact that people may change their behavior in response to weather—for example, staying indoor or taking days off when outdoor temperatures are high (see Discussion section below).

### Curve fitting

#### Sinusoidal functions for monthly population changes.

To estimate seasonal city-scale population changes and understand their correlation with temperature, we fit each city’s monthly population and mean temperature profiles (within the UrbClim model domain) with a sinusoidal function, as commonly done to describe the seasonal variation of climate variables (e.g., [52,53]). We use cosine functions to model the gradual and periodic change in monthly population during a year as this could serve as a simple model for other cities where monthly-varying population data are not available. Specifically, monthly population and temperature cycles are fitted with a cosine function of a 12-month period, i.e.,

$$P_{t}=A\cdot \cos\left(\frac{2\pi }{12}\cdot (t+b)\right)+c$$ (1)

where *P*_*t*_ is the total population of the city during month *t*. Eq 1 has three free parameters: *A* (amplitude) related to how strong the seasonal fluctuation is, *b* (phase shift in the unit of months) relates to when the population highs/lows occur, and *c* (baseline population) relates to the average population of the city throughout the year. The function is fitted to 12 monthly population counts in each city using non-linear least squares. As the population highest/lowest points often occur in the summer months (June to August) across most cities, the value of *b* is bounded within 0 to −2 for the fitting (*b* = 0 or –2 means that the highest/lowest points occur in June or August, respectively). The bounds are not specified when fitting monthly temperature variation. We use the curve_fit() function in Python package scipy.optimize.

#### Linear and exponential fittings.

The linear fitting, with the calculation of slopes and R-squared in Fig 3, is conducted using the linregress() function in the Python package scipy.stats. The exponential fitting and the fitting statistics in Fig 4 are performed by the curve_fit() function in the Python package scipy.optimize.

### Temperature exposure and risk

Temperature-mortality exposure-response functions (ERFs) are typically J- or U-shaped curves relating temperature to the relative risk (RR) of mortality and depend on physiological factors such as age and acclimatisation to a particular climate, as well as socioeconomic factors, such as building insulation and access to air conditioning [8,54]. Relative risk indicates the ratio of risk among the exposed group (i.e., being exposed to non-optimal temperature) to the non-exposed group [55,56], against temperature percentiles. The temperature percentile associated to the lowest RR (i.e., *RR*=1) is the minimum mortality percentile (MMP) and its corresponding temperature is the minimum mortality temperature (MMT), which indicates the optimal temperature for the population [57]. As such, there is a large variation of MMT values across cities around the world [54,57], which allows us to translate changes in exposure into more tangible, city-specific, considerations.

ERFs specific to different age groups (20-44, 45-64, 65-74, 75-84, and 85+) are obtained for each city as detailed in Masselot et al. [8] and Huang et al. [19]. These ERFs are derived using daily mortality time series from the multi-country multi-city (MCC) collaborative research network and 9km resolution daily mean temperature data from ERA5. Huang et al. [19] further transformed the percentile-based ERFs into functions based on each city’s UrbClim domain average daily temperature from 2008 to 2017. These relationships quantify the mean RR for each city- and age-specific population, assuming applicability to the entire population within each age group in each city, regardless of individual differences in location, exposure, or vulnerability. In our study, we specifically select the ERFs for the age group of 45 to 65 years, as this group constitutes the working-age population (15-64 years as defined by Organisation for Economic Co-operation and Development, OECD), and thus is more likely to commute daily than older age groups. The choice of the age group is deemed reasonable for our study, given that ERFs for younger age groups (i.e., 20-45) have higher uncertainties [8]. The main merit of using city-specific MMTs and ERFs is that it considers population acclimation and socioeconomic factors specific to each city instead of employing a constant temperature threshold for every location as commonly done in previous studies (e.g., [30,58]).

We formulate our indices for exposure and risk in alignment with the definitions provided by the Intergovernmental Panel on Climate Change (IPCC) convention [59]. According to the IPCC, exposure is defined as “the presence of people...in places and settings that could be adversely affected.” In the context of our study, deviation in temperature from MMT implies an increasing risk. Thus, we use changes in population above/below MMT to quantify the change in heat/cold exposure. The IPCC convention defines risk as “the potential for adverse consequences”, which would be the human mortality risk in our study, as represented by RR as the risk index [19].

### Rescaling distance and population

To analyze temperature and population variations as a function of distance to the city center for different cities, we follow an established approach to rescale distance *r* (Eq 2) and population *P* (Eq 3) based on the population of the largest city (Eq 4) (in our study this is represented by Paris daytime population in April) [60]. The rescaling procedure is as follows:

$$r^{\prime }=\frac{r}{k}$$ (2)

$$P^{\prime }(r^{\prime })=\frac{P}{k}$$ (3)

$$k={\frac{P_{city}}{P_{Paris}}}^{1/3}$$ (4)

where $r^{\prime }$ and $P^{\prime }$ are the rescaled distance and population, respectively, and *k* is the rescaling parameter. Following the rescaling, we construct the radial profile by calculating the average temperature and population of the grid cells located between 1 ∼ 1.5 km, 1.5 km ∼ 2 km, ... up to 29.5 ∼ 30 km. The distance is calculated from the geographical center of the UrbClim city domain, which considers the administrative boundaries and immediate surroundings of each city. This procedure allows us to obtain the average temperature and population along the radial distance at 0.5 km resolution in such a way that cities with different sizes can be compared to each other.

Note that we estimate the radial profiles of population change and heat exposure using the geographical center of the UrbClim domain. This choice is different from using the location of the town hall as used by Batista e Silva et al. [17], which partially explains why our radial profiles of population are not identical to their study. Yet, Lemoy and Caruso et al. [60] showed that the population radial profiles are quite insensitive to the precise location of the city center, thus confirming the validity of our choice. Other differences may come from the slightly different subsets of cities (31 versus 27 cities in our study, see Fig 4a–4c). The resultant radial profiles have higher fluctuations at short distances than at longer distances (Fig 4b, 4c) probably because there are fewer data points at short distances. In addition, some cities with low population do not have data at the shortest rescaled distances because their distances become larger after rescaling. It should also be noted that not all cities are monocentric [17], and although we excluded water surfaces (e.g., sea or rivers) in the radial profiles, some cities have clear geographical constrains on land (e.g., forests or hills) such as Athens and Nice which partly affect the observed profiles (see next section).

## Results

### Daily and seasonal urban population dynamics

**Fig 1:**
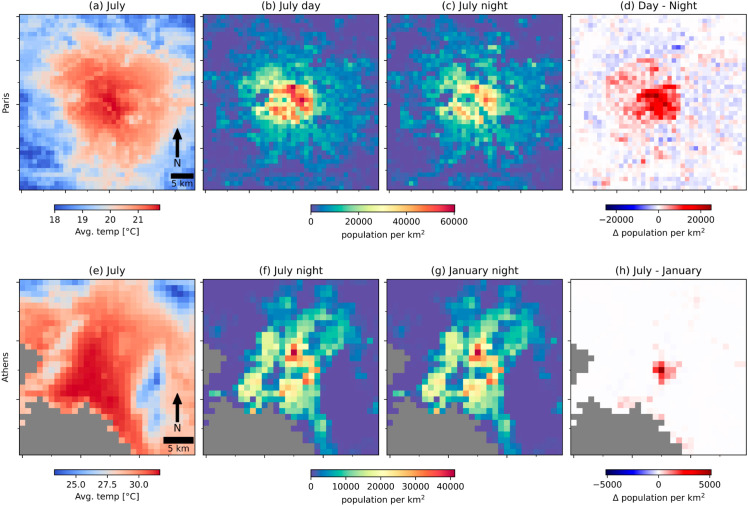
Spatial distribution of air temperature and population in Paris and Athens. Temperature maps show the average temperature in July 2010-2012 (a & e). The population maps of Paris show the diurnal changes between day and night in July (b-d). The maps of Athens show the seasonal nighttime population difference between July and January (f-h).

On a diurnal basis, the daytime population (*P*_*day*_) of an urban area is generally higher than the nighttime population (*P*_*night*_) because people commute to cities for daytime activities as we illustrated for the city of Paris in Fig 1b–1d. On a seasonal basis, human mobility mostly depends on holiday calendars and vacation destinations. It is common that warm, seaside, and culturally-rich cities (like Athens, for example) draw more tourists than its outbound residents in the summer, resulting in an increased population (Fig 1f–1h). In both cases, the locations where population increases during the day/summer is often correlated with the locations where background temperature is the highest (Fig 1 and S4 Fig). This can be explained by the fact that people are generally drawn to more urbanized areas with more facilities and services, which typically have higher UHI intensities. This suggests that diurnal and seasonal population mobility may exacerbate heat exposure but the generality of their mobility-exposure patterns has yet to be demonstrated.

**Fig 2:**
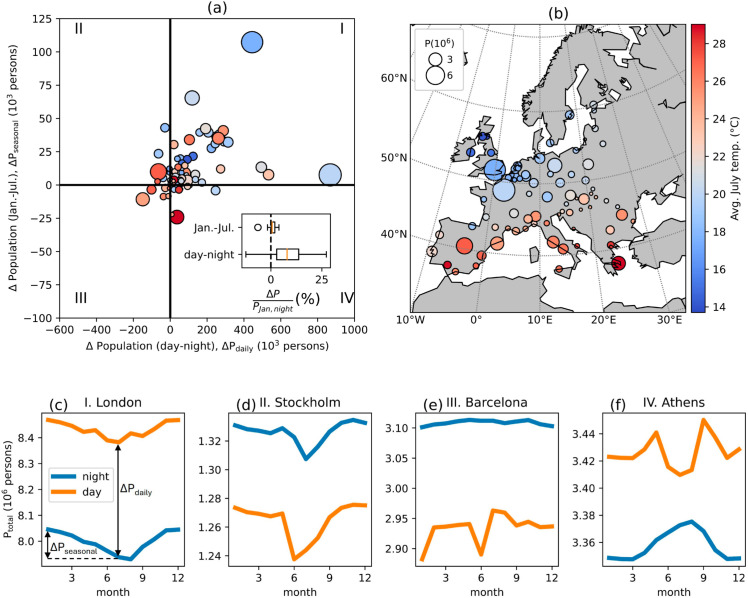
Daily and seasonal mobility in European cities. Four types of diurnal and seasonal population mobility patterns in 80 European cities (a). Daily mobility is calculated as the difference between daytime and nighttime population in July. Seasonal mobility is calculated as the difference between nighttime population in July and January. Nighttime population in January is used for plotting the size of the circles in (a) and (b). Average July temperatures from 2010-2012 are represented by the color of the circles. The boxplot of daily and seasonal population changes in terms of percentage in all the selected cities is shown in the inset in (a). The boxes in the boxplot shows the median and the first and third quartiles, whiskers the minimum/maximum value within 1.5 times the interquartile range from the first/third quartiles, and dots the outliers. Monthly total daytime and nighttime population estimates for four selected cities (London, Stockholm, Barcelona, Athens) representing four types of mobility patterns are shown in panels (c) to (f).

The analysis of 80 European cities reveals the existence of distinct patterns of daily and seasonal population variations, which can be classified into four groups based on the net change in population (below or above zero), as illustrated by the quadrants in Fig 2. These quadrants are defined by the cities’ population trends, distinguishing between higher population during daytime or nighttime and higher population in the summer (July) or winter (January) (Fig 2a). To highlight the diversity of population variations observed across Europe, daytime and nighttime monthly population counts for four selected cities (one per quadrant) are also shown in Fig 2c–2f. Here we define $\Delta P_{daily}$ as the city’s total population change between nighttime and daytime in July, and $\Delta P_{seasonal}$ as the difference in nighttime population between July and January. These cities show that although *P*_*day*_ and *P*_*night*_ fluctuate throughout the year, the sign of $\Delta P_{daily}$ remains consistent, and its magnitude is largely stable. The majority of the selected European cities (56 out of 80 cities) have higher population during daytime and winter (the first quadrant in Fig 2a), so both their $\Delta P_{daily}$ and $\Delta P_{seasonal}$ are positive (as in the case of London, Fig 2c). As expected, most cities (66 in the I and IV quadrants in Fig 2a) have a higher population during daytime than at night, implying that using nighttime population would underestimate actual heat exposure. However, a few cities (e.g., Barcelona, Madrid, Stockholm, and 11 others in the II and III quadrants in Fig 2a) exhibit a higher population at night, as noted previously by Batista e Silva et al. [17] and attributed to a possible underestimation of daytime population in some cities. These trends could also be explained by city-specific characteristics (e.g., the presence of commercial and industrial areas at the urban fringe) but, given the uncertainty associated with the population estimates of these cities, we restrict our analysis of daily mobility to the urban areas in quadrants I and IV only. Yet, for the sake of completeness, results for cities in group II and III are also shown in the Supporting Information. Regarding the seasonal variations, most of the selected cities have lower population in the summer (64 cities belonging to the I and II quadrants in Fig 2a), mainly because residents traveling outwards for summer holidays exceed the incoming population. Cities with higher population during summer are usually warm seaside cities in Southern Europe which are typical destinations for summer holidays, e.g., cities on the Spanish coastline (Barcelona, Malaga), the French Riviera (Nice, Marseille), and in Greece (Athens, Thessaloniki) and 8 others in the III and IV quadrants in Fig 2a. These cities experience high summer temperatures (Fig 2b), and the influx of more people during the summer exacerbates heat exposure. Overall, the day-night population difference in July can be up to 27% (Budapest), while the magnitude of seasonal variation is up to 6% (Varna) (the inset plot in Fig 2a).

**Fig 3:**
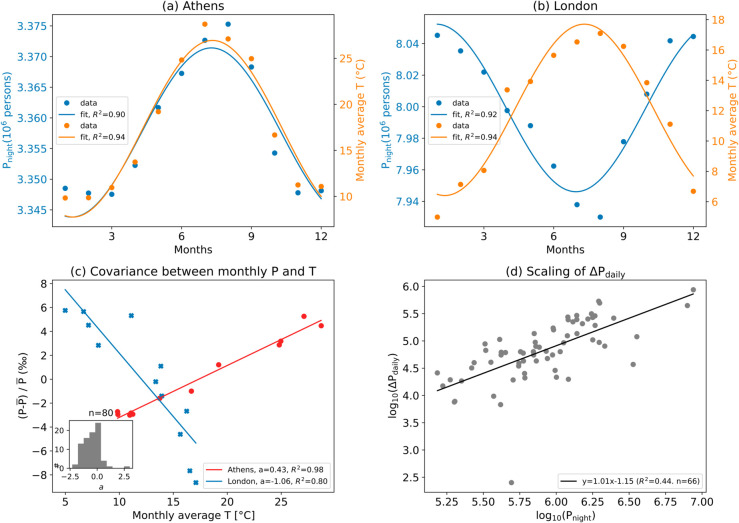
Covariation of urban population and climate. Seasonal cycle of monthly temperature and nighttime population fitted to sinusoidal functions in Athens (a) and London (b). Fitted linear relationships between monthly population (plotted as relative deviations from the mean value $\bar{P}$) and monthly average temperature in 2011 (c). Insets in (c) show the distribution of slopes of the fitting lines for the 80 cities. Scaling of the population daily mobility as a function of city size for the 66 cities with higher daytime than nighttime population (d). Scaling relationship of the cities with higher nighttime than daytime population (14 cities) is shown in S3 Fig.

The analysis of seasonal mobility reveals that population in part of the 80 European cities have a clear seasonal cycle with a period of about 1 year and the peak either during summer (e.g., July) or winter (e.g., January), especially for nighttime population because of its more gradual variation across months (see Fig 2e, 2f). In Fig 3 Athens and London are shown as two examples of cities with clear sinusoidal cycles (see Materials and Methods), but contrasting seasonal mobility patterns. The population in Athens increases steadily through spring, peaks in the summer, and gradually declines back to spring-level over the autumn and winter. The population peak coincides with the temperature peak in the summer, which thus increases heat exposure (Fig 3a). In contrast, London’s population decreases in spring, hits a summer low, and rebounds in autumn and winter. Hence, being population variations out-of-phase with the temperature cycle, heat exposure in summer is reduced (Fig 3b). The *R*^2^ values of the fitted sinusoidal functions for monthly *P*_*night*_ are above 0.5 for 62 out of 80 cities, showing generally good performances, while for monthly *P*_*day*_ only 39 cities show *R*^2^ above 0.5. The *R*^2^ values of the fitted sinusoidal curves for all 80 cities is shown in S2 Fig.

Given this seasonality, the relationship between monthly average temperature and population in the selected cities is well-represented by a linear function (Fig 3c). Specifically, the linear fitting allows to clearly distinguish the two main trends discussed earlier: cities like Athens where population fluctuations are in phase with climate seasonality (i.e., both population and temperature peak during summer) exhibit a linear trend with positive slopes while cities like London where seasonal changes in population are out of phase with temperature have a negative slope, which is associated with a decrease in heat exposure. The fitted slopes range from 3 ‰ to −2.3 ‰. Varna, a seaside city on Bulgaria’s Black Sea coast, has the highest fitted slope (∼3.0 ‰/${\;\;}^{\circ }C$) followed by Nice (∼1.0 ‰/${\;\;}^{\circ }C$) on the French Riviera and Malaga (∼0.7 ‰/${\;\;}^{\circ }C$) on the Spanish coastline. Conversely, cities in colder regions like Rotterdam (∼−2.3 ‰/${\;\;}^{\circ }C$) and Utrecht (∼−2.2 ‰/${\;\;}^{\circ }C$) have the most negative slopes. The fitted lines of 60 cities give *R*^2^ values above 0.5, proving overall good fits for most cities. The *R*^2^ values all 80 cities are shown in S2 Fig.

Daily mobility patterns also show a significant regularity across cities, as revealed by the scaling of daily population changes with city size (Fig 3d). For the 66 cities with $\Delta P_{daily}>0$ (in quadrants I and IV in Fig 2a), a scaling law was found between $\Delta P_{daily}$ and nighttime population: $\Delta P_{daily}∼P_{night}^{\beta }$ with the exponent *β* close to unity (Fig 3d). In other words, the net incoming daytime population is proportional to the city’s nighttime population, which is in line with gravitational theories of human mobility (see Discussion section).

### Intra-urban population distribution and temperature exposure

**Fig 4:**
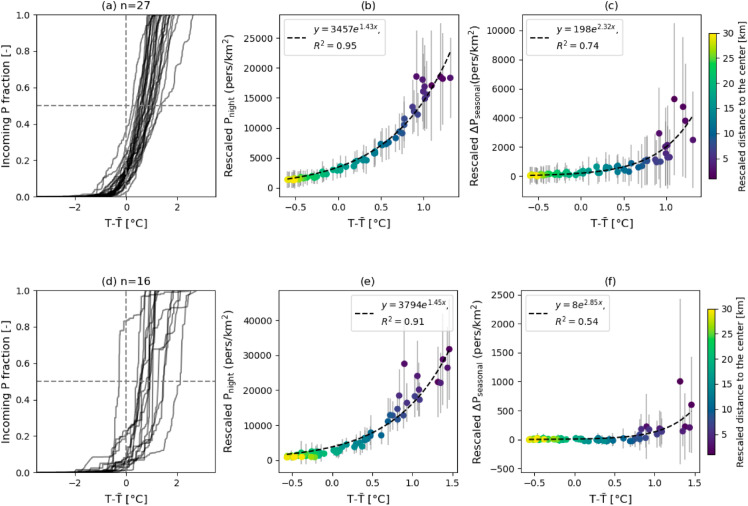
Intra-urban variations in temperature exposure. Cumulative incoming population fractions from low to high temperatures across 27 large European cities over 1 million population (a) and 16 cities which increase population in the summer (d). Temperature in different cities are normalized by subtracting the city’s mean temperature over its UrbClim domain. Average nighttime population of the subsets of cities (b) & (e), $\Delta P_{daily}$ (c), and $\Delta P_{seasonal}$ (f) against average temperature minus the city’s mean along the rescaled radial distances (see Methods) at 0.5-km resolution from the UrbClim domain geographical center. Standard deviations across the cites are shown as the error bars in b,c,e,f. Radial profiles in the winter are shown in S6 Fig.

The previous sections focused on net population change at the city scale without considering the intra-city population heterogeneity. Yet, we find that both residential and incoming population within a city are highly concentrated at locations where temperature is above the mean, which thus largely modifies exposure. In Fig 4a, the percentage of incoming population (only considering the grid cells with positive $\Delta P_{daily}$) cumulated from low to high temperatures is shown for all the cities with more than 1 million nighttime population in quadrant I and IV (27 cities, see Fig 2a). Results show that 85.5% (IQR:89.8-82.9%) of the incoming population visits locations with temperatures above the local average ($\bar{T}$), with the highest ratio in Dublin (97.3%) and the lowest ratio in Lisbon (63.7%). Due to the different local microclimatic conditions, the temperature ranges are different across cities, with the highest $T\;-\;\bar{T}$ in Athens (2.8 ${\;\;}^{\circ }C$) and the lowest in Warsaw (0.96 ${\;\;}^{\circ }C$). Clustering of incoming population at warmer urban locations is also observed in the 16 cities which gain population in the summer on a seasonal basis (cities in quadrants III and IV in Fig 2a) (Fig 4d).

The strong correlation between population and temperature stems from their common increasing trends towards the city center. To compare the temperature across the cities, we normalize it by subtracting the city’s average temperature. The radial profiles of daytime population and temperature show remarkably similar increasing trends towards the centers across the 27 cities analyzed (S4 Fig). Averaging across the cities, both nighttime population and $\Delta P_{daily}$ follow a relationship that is exponentially-increasing towards locations with warmer temperatures, which are also closer to the geographical center of the cities (Fig 4). In other words, the daytime population inflow becomes significantly larger closer to the warmer and more central locations, implying a surge in mobility-induced heat exposure in city centers. The seasonal mobility in the 16 cities in quadrants III and IV (see Fig 2a) causes similar results, although with less intensity (Fig 4e, 4f). In winter, the same correlation between population and temperature is observed (S5 Fig), which increases cold exposure (but decreases risk, see next sections).

### Mobility effects on temperature exposure and risk

**Fig 5:**
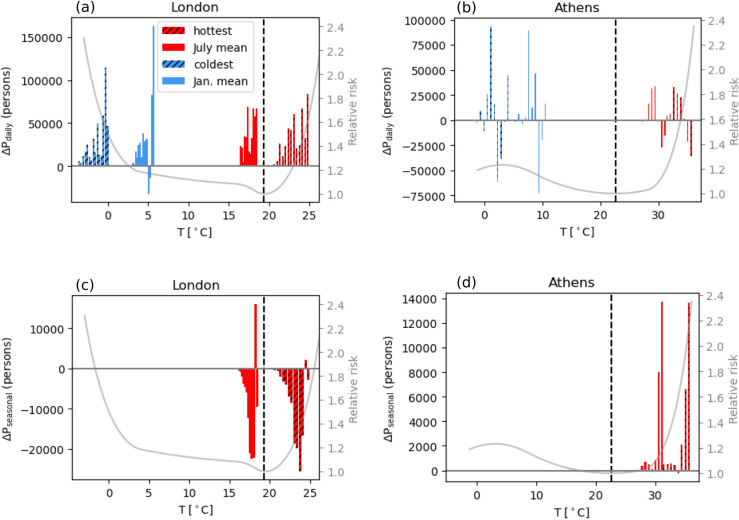
Mobility and heat risk. Impact of daily (a-b) and seasonal (c–d) population mobility on heat exposure in London (a-c) and Athens (b–d). The gray curve is the city’s ERF for the age group 45–65 with the dashed black line marking the MMT [19]. Daily population difference is calculated by day minus night population in July. Seasonal population difference is calculated as nighttime population difference between January and July. The hatched histograms show average temperatures during the warmest/coldest 3% days in July/January 2010 to 2012. The histograms without hatching are plotted with the monthly average temperature of July/January during the same years.

The exponential relationship between population variations (both diurnal and seasonal) and temperature means that commuters and tourists are exposed, on average, to temperatures higher than the city’s average. However, how the temperature-related risk changes depends on the population’s response to that temperature. Health outcomes are related to a variety of factors (e.g., age, diet, socio-economic conditions) but, as a first approximation, we can compare intra-urban variations in temperature exposure with city specific temperature-mortality exposure-response functions (ERF) and assess deviations from the minimum mortality temperature (MMT) (see Materials and Methods). As an example, we illustrate this approach in Fig 5 for the cities of London and Athens. During the hottest days of July, positive $\Delta P_{daily}$ in London occurs at locations with temperature above the MMT, which points to an increase in heat risk (Fig 5a). On the other hand, during an average summer day, temperatures across London are near its MMT, so even though $\Delta P_{daily}$ is positive, it does not alter heat risk significantly (rather, it provides a slighty protective effect). During winter, $\Delta P_{daily,Jan}$ (i.e., day-night differences during January) increases exposure as the number of people in the city during daytime is higher but, considering that the population is commuting from colder rural areas towards a warmer city center (due to UHI effect), mobility is likely reducing cold-related risks (see the next and Discussion sections). In contrast to the strong correlation between $\Delta P_{daily}$ and temperature in London, the correlation in Athens is weaker (see Fig 1, but daily mobility still increases heat/cold exposure overall (Fig 5b). On seasonal timescales, mobility in London reduces population across the entire urban area (i.e., across the entire range of urban temperatures), so heat exposure in July is reduced (Fig 5c). The contrary happens in Athens, where population is higher during summer and clusters in regions with temperatures associated with higher heat-risk (Fig 5d).

**Fig 6:**
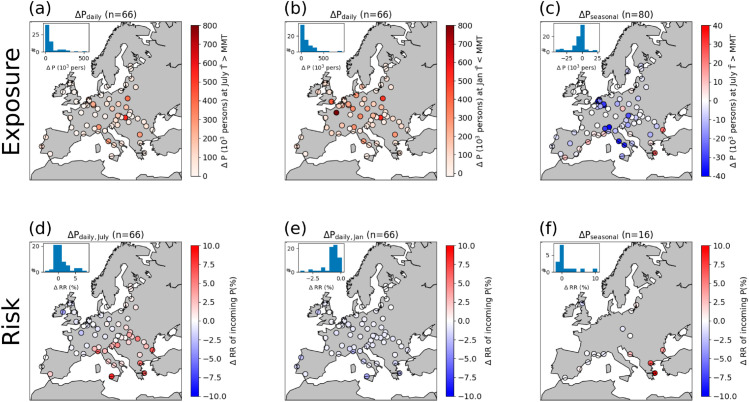
Mobility-induced temperature exposure and risk. Daily mobility modifies heat/cold exposure to temperatures higher/lower than MMT in July/January (a, b). Seasonal mobility modifies heat exposure in July (c). Difference between RR using incoming population-weighted temperatures and city’s mean temperatures when daily (d,e) or seasonal (f) mobility is considered. The temperature distribution is from the July (a,c,d,f)/January (b,e) average temperature 2010-2012. RR is derived using city’s specific ERF for the age group 45-65. Seasonal population difference is calculated as nighttime population difference between July and January. (a,b,d,e) show the 66 cities in quadrants I and IV in Fig 2 with increased population during daytime and (f) shows 16 cities in quadrants III and IV with increased population during summer.

Applying MMT as the threshold for heat/cold exposure for all cities, we find that daily mobility, by bringing population toward city centers (groups I and IV in Fig 2a), increases city-total heat exposure in summer (July) and cold exposure in winter (January) at a comparable magnitude (Fig 6a, 6b). Heat exposure does not increase in some cities in North-Western Europe because their July average temperature is below their MMTs (e.g., as shown by London in Fig 5a). However, during heat extremes, daily mobility increases heat exposures in all the cities, because temperatures are above the MMTs threshold (S9 Fig). On average, positive $\Delta P_{daily}$ increases heat exposure (population above MMT) by 70,984 people (IQR: 6’135-77’115) during a day in July (7.8% with IQR: 1.0-13.0% in terms of July nighttime population). Budapest has the highest excess heat exposure (531’510 or 27.1%) on an average summer day, followed by some cities in Eastern Europe. Daily mobility increases cold exposure in winter (population below MMT) by on average 129’280 people (IQR: 32’674-174’719) (11.5%, IQR: 4.9-17.2%) with the largest increase observed in Paris (899,502) (or Bratislava in terms of percentage, 32.3%). On seasonal time scales, mobility has a weaker influence on temperature exposure, and it can either increase or reduce heat exposure depending on population flowing out or into cities during the summer (Fig 6c). Heat exposure is only increased in the 16 cities (in III and IV in Fig 2a) that attract population in the summer and is reduced in all the others. Adjusted exposures in terms of percentages are shown in S10 Fig.

To quantify the average temperature and relative risk (RR) experienced by the incoming population, we compare RR at the incoming population-weighted temperature among only the grid cells with positive ΔP with the RR at city’s mean temperature. Population-weighted temperature is calculated as $\langle T\rangle =\Sigma T_{i}P_{i}/\Sigma P_{i}$ where *i* represents the grid cells with positive ΔP. A similar method applying population-weighted temperature to ERFs to assess heat risk was done in a previous study [18]. Across the 66 cities with positive $\Delta P_{daily}$, the incoming population-weighted temperature is 0.71 ${\;\;}^{\circ }C$ (IQR: 0.44 - 0.89 ${\;\;}^{\circ }C$) above the cities’ averages (not shown). The increased temperatures modify the RR according to the city-specific ERF, so increased temperature does not necessarily translate into increased RR, depending on the MMT and the shape of the ERF. Fig 6d reveals a striking South-East to North-West gradient in such changes in temperature-related risks, with mobility increasing the RR borne by incoming population only in cities in South-Eastern Europe, while RR is reduced in North-Western European cities. The reason is that the average temperatures in July are below MMTs for cities in North-Western Europe, so being exposed to higher temperature actually provides a health benefit. In winter (January), temperatures across European cities are below their respective MMTs, so incoming population benefits from the protective effect of UHIs against cold, showing an average change in RR of −0.76% (IQR: −0.42%–0.84%) (Fig 6e). Finally, for the cities which gain population during summer, the RR of the seasonal incoming population (considering July average temperature) is increased by up to 10.4% in Athens, followed by Thessaloniki (6.7%) and other Mediterranean cities (Fig 6f). RR of the incoming population is slightly reduced in some cities (e.g., Edinburgh) where temperature is below the MMT on an average summer day, but it raises largely during heat extremes (S12 Fig).

## Discussion

Our study analyzes seasonal and daily mobility patterns across 80 European cities and quantifies the relation between mobility-modified population and temperature distributions as well as their dependence on the distance from the city center. Mobility-induced excessive temperature exposure and risk are also estimated by combining city-specific ERFs with population-temperature distributions. The 80 European cities show diverse daily and seasonal mobility patterns depending on the population behaviors and background climates. Although seasonal mobility generally has a smaller magnitude than daily mobility (Fig 2a), its effect on heat risk should not be overlooked, especially for hot touristic destinations which gain population during the summer. Our analysis of monthly population variations demonstrates that human sub-annual traveling behavior follows a regular and predictable seasonal cycle, which is related to seasonal variations of climate as already documented for human physical activities [61] and crime rates [62]. Specifically, we find that seasonal mobility in most cities (but not all of them, see S2 Fig) can be described by sinusoidal functions which are either in-phase or out-of-phase with the seasonality of air temperature. We also find a scaling relationship between city size and daily changes in population, with an exponent β≃1 (Fig 3d). Such scaling relationship can be explained by considering the gravitational law of aggregated population flows (*T*_*ij*_) between two locations *i* and *j*. This law, which can be expressed as $T_{ij}=kP_{j}P_{i}f(r_{ij})$ [63,64], states that the inter-city flows (*T*_*ij*_) are proportional to the product of the populations at origin and destination and a function *f*(*r*_*ij*_) that decays with the distance between them. If we consider an entire city as destination *j*, *P*_*j*_ then represents its total residential population, *P*_*i*_ is the population at any location *i* outside the city, *r*_*ij*_ is the distance between *i* and *j*, *k* is a constant, and *f*(*r*_*ij*_) is typically an inverse power-law of *r*_*ij*_. Because *k* and *P*_*j*_ are constants, summing over all the possible origin locations *i*, leads to $\sum_{i=0}^{n}T_{ij}=kP_{j}\sum_{i=0}^{n}P_{i}f(r_{ij})$ (similar to the derivation in [65]) which implies that $\Delta P_{daily}∼\sum_{i=0}^{n}T_{ij}∼P_{j}$, thus confirming the linear scaling observed. Such simple scaling arguments, in combination with the sinusoidal functions describing the seasonality of nighttime population, can provide preliminary estimates of expected net incoming population during different periods of the year, thus improving traditional approaches based on population census only. As such, this knowledge can be of interest not only for environmental exposure assessment, but also for public transportation system, emergency evacuation planning, epidemic modelling, and urban planning.

For intra-urban distributions of heat exposure, our study confirms previous evidence showing that UHIs and daytime population exponentially increase towards city centers (some studies proposed power-law relationships, depending on the considered urban extent [66]), and further discovers that the relationship between mobility-adjusted population and temperature is also well described by an exponential function. These findings confirm and quantify the co-variation of urban-induced warming and daily incoming population, thus pointing to central urban areas as hot-spots of population exposure to urban temperatures.

Our study shows that similar mobility behaviors (i.e., increasing urban population during daytime) can have different impacts on temperature exposure and risk, depending on each city’s background climate and population vulnerability to heat. We show that the MMT can be used as a temperature threshold to quantify and compare heat/cold exposure across different cities and climates. A major finding here is that daily mobility hardly modifies heat exposure/risk for North-Western European cities during average summer days, as temperature is often below or near to their respective MMTs (Fig 6a, 6d). One reason is that minimum mortality percentile (MMP) increases generally from low altitude to high altitude, ranging from 68th percentile in some Spanish cities to 96th percentile in several cities in the UK and Ireland [8]. This means that average summer temperatures are likely above the MMTs in Southern European cities but under or near the MMTs in Northern European cities. Hence, exposure (to temperature above MMT) and the associated risk does not increase despite a net positive incoming population. A key take-home message from these results (see Fig 6a, 6d and S9 Fig) is that changing daily commuting behavior solely during heat extremes can largely alleviate mobility-induced heat exposure/risk in North-western European cities, while interventions are required throughout the summer for cities in the other European regions. Therefore, changing commuting and traveling behavior should be considered as a flexible measure to mitigate temperature-related health risks according to the season. This approach can complement standard UHI mitigation methods which are effective in reducing heat but may inadvertently reduce the protective effects of urban warming against cold weather [43].

Although the daily incoming population-weighted temperature is, on average, just about 0.71 ${\;\;}^{\circ }C$ above the city’s mean, this magnitude of change in temperature can lead to significant shift in RR (Fig 6d). As shown in Fig 6d, Southern and Eastern European cities have larger increases in RR, which is in line with the higher temperature-related mortality risk observed elsewhere in Southern and Eastern Europe [8]. Many Central and Eastern European cities also experience higher heat exposure due to daily mobility (Fig 6a). Hence, heat risk in these regions is exacerbated by a combination of lower MMP, higher RR as temperature rises, and high daytime population inflows. Certain Southern European cities, especially in Greece, experience elevated heat exposure and risk resulting from both daily and seasonal mobility and particular attention is thus required for effective heat adaptation.

It is important to note, however, that our study does not consider a variety of factors which may play an important role for the actual exposure of urban population to extreme temperatures. First, the population mobility behavior is not coupled with urban climate, as we just studied the correlation between two independent population and temperature datasets (see Materials and Methods). Second, we assessed temperature exposure at the city rather than individual scale. For example Fig 5 shows the change of total temperature exposure in London, but it does not account for the origins/destinations of incoming/outgoing individuals. During summer, outgoing individuals may go to warmer Southern European cities, so their individual heat exposures are likely to increase while the total exposure in London reduces. In fact, individual heat exposure depends on socioeconomic factors [32,67,68], age [8,19], commute modes and pathways [68–71], as well as indoor/outdoor differences in microclimate [72]. Such a detailed assessment is beyond the scope of this study but addressing the complexity of actual human exposure to temperature is a key direction for future work.

Also, it should be noted that the ERFs used here [19] represent city-scale health outcomes, but a recent research in the UK showed that the shape of ERFs (and the associated MMT values) vary at the intra-urban level [73]. Therefore, the heat risk calculated here is based on city-average sensitivity to temperature and should be seen as a first quantification instead of a precise estimate. Besides, certain population groups may perceive differently and be more/less sensitive to extreme temperatures than predicted by the cities’ 45-to-65-year-old averaged curves used here. Also, we did not consider that heat effects of a given temperature is larger in spring or early summer due to lower acclimation [74]. Another limitation of the ERFs is the lack of consideration of relative humidity, while research has found that the compound occurrence of high air temperature and relative humidity can enhance mortality risks [9]. We also acknowledge that there are various temperature thresholds used in the literature other than MMT, for instance daily wet-bulb globe temperature of 30°C [30], daily maximum air temperature exceeding 25 or 30°C [58], daily maximum air temperature exceeding 35°C or 40°C [26,27,75], although they do not account for variability in population long-term adaptation to local climate.

Regarding mobility, we acknowledge that our approach is not able to distinguish if the population changes are caused by mobility within or from outside the urban domains due to the constraint of the population dataset (see Materials and Methods). Hence, future work should focus on more detailed observational (e.g., GPS tracking) and modeling (e.g., agent-based) approaches. Besides, random, irregular, or low-frequency mobility behaviors are not captured; for instance, changes caused by extreme weather, lockdowns, natural disasters, or short festivals and events. Also, the increased summer population in warmer cities are partly composed of tourists from colder countries, who are less acclimated to high temperatures. Hence, using local MMT values for (foreign) tourists likely leads to an underestimation of the actual risk thresholds. In general, it is worth noting that human travel behavior may change over time and be influenced by climate change [76]. For example, a temperature increase of 3°C or 4°C is projected to reduce summer tourists by almost 10% in southern Europe while raise the tourism demand for northern Europe by 5% [77]. Daily mobility may also change in time and space with changes in behavior (e.g., post-COVID changes in teleworking frequency [78]) and/or urban development. For example, small cities may keep attracting new residents and companies in the city center, while population in larger cities may spread out from the central areas to the outskirts by following the development of transport networks [79]. Hence, population dynamics are constantly evolving, thus limiting any extrapolation of the results here (which are based on historical observations) to future conditions.

## Conclusion

Despite its significance in cities, mobility is often an overlooked factor in existing studies on urban heat exposure and risk. Using urban climate simulations, dynamic population data, and state-of-the-art temperature-mortality exposure-response relationships from 80 European cities, our study highlights the key role of human daily/seasonal mobility in assessing heat exposure and risk in urban areas. In general, the observed increase in temperature exposure caused by mobility suggests that adjusting commuting and traveling behaviors can provide an important contribution to future improvements in thermal well-being and health. Intervention such as implementing days off, adjusting working hours, temporarily closing public services, schools, or outdoor tourist attractions during extreme temperature events can mitigate hazardous exposure. A proper quantification of daily and seasonal mobility is therefore essential for authorities to implement these adaptation measures, targeting exposure hotspots and peak periods (i.e., where and when the health risk for the population is the highest).

## Supporting information

S1 FigCorrelation between $\Delta P_{daily}$ and average temperature.Correlation between $\Delta P_{daily}$ and average temperature in July 2010-2012 in Paris (a). Correlation between $\Delta P_{seasonal}$ and average temperature in July 2010-2012 in Athens (b). Each dot shows data of a 1-km grid from the distribution.(PNG)

S2 Fig *R*^2^ of the fitted functions. *R*^2^ of the fitted cosine function to monthly *P*_*night*_ (a), *P*_*day*_ (b), and monthly average temperature (c). *R*^2^ of the linear fitting between monthly average population and nighttime population (d).
(PNG)

S3 FigScaling relationship of the cities with higher nighttime than daytime population (14 cities).Same figure as Fig 3d but with the 14 cities in quadrants II and III in Fig 2a that has higher nighttime population than daytime population. Noted that a negative sign is in front of $\Delta P_{daily}$ in order to keep the values positive before taking logarithm.(PNG)

S4 Fig Radial population profiles in the summer. Average rescaled *P*_*day*_ against temperature along the radial profile across the 27 largest European cities (above 1 million) in quadrant I and II in Fig 2a in July. The temperature distribution is from the July average temperature from 2010 to 2012. Temperatures in each city are normalized by subtracting the city’s UrbClim domain’s average temperature.
(PNG)

S5 Fig Radial population profiles in the winter. Average rescaled *P*_*day*_ against temperature along the radial profile across the 27 largest European cities (above 1 million) in quadrant I and II in Fig 2a in January. The temperature distribution is from the January average temperature from 2010 to 2012. Temperatures in each city are normalized by subtracting the city’s UrbClim domain’s average temperature.
(PNG)

S6 Fig Intra-urban variations in temperature exposure in the winter. Same figure as Fig 4, but in January. The temperature distribution is from the January average temperature from 2010 to 2012.
(PNG)

S7 Fig Radial profiles of the 14 cities with higher nighttime population. Same figure as Fig 4, but with the 14 cities in quadrants II and III in Fig 2a that show lager nighttime population than daytime population.
(PNG)

S8 Fig Spatial distribution of daytime and nighttime population in Madrid. Same as Fig 5a but with Madrid where the dataset [17] shows higher nighttime than daytime population. Daily population mobility changes human heat exposure in Madrid where nighttime population is higher than daytime population. The gray curve is the city’s ERF for the age group 45-65 with the dashed black line marked the MMT. Daily population difference is calculated by daytime minus nighttime population in July. The hatched histograms are plotted with temperature averaged over the warmest/coldest 3% days in July/January from 2010 to 2012. The histograms without hatching are plotted with the monthly average temperature of July/January over the same years.
(PNG)

S9 Fig Daily mobility-induced temperature exposure maps during heat extremes. Same figure as Fig 6a but with the average temperature distribution of the hottest 3% days in July 2010-2012. Change in heat exposure is positive in all the cities.
(PNG)

S10 Fig Mobility-induced temperature exposure maps in terms of percentage. Same as Fig 6a–6c but with adjusted exposure in terms of percentage of July nighttime population (a,c) or January nighttime population (b) for all 80 cities.
(PNG)

S11 Fig Mobility-induced temperature exposure maps of all cities. Same as Fig 6a, 6b but with all the 80 cities including the 14 cities where the dataset [17] shows higher nighttime than daytime population.
(PNG)

S12 FigSeasonal mobility-induced temperature exposure maps during heat extremes.Same figure as Fig 6f but with the average temperature distribution of the hottest 3% days in July 2010-2012. Heat risk increases in all the cities except Malaga where population is estimated to move to the beach on the colder outskirts of the city. The increase in RR is significantly higher than the values shown in Fig 6f where the July monthly average temperature was considered. Noted that for 2 cities (Thessaloniki and Klaipeda) ΔRR is only calculated by taking the maximum RR on the warmer side of the ERF as estimates because the incoming population-weighted temperatures are above the temperature range in the UrbClim temperature-derived ERFs by Huang et al. (2023) [19]. Therefore, the actual ΔRR may be even higher.(PNG)
