# Supplementary figures and images for: Daily and seasonal human mobility modulates temperature exposure in European cities
Source: PLoS One. 2025 Sep 3;20(9):e0330912. doi: 10.1371/journal.pone.0330912 (PMC12407427; doi:10.1371/journal.pone.0330912)

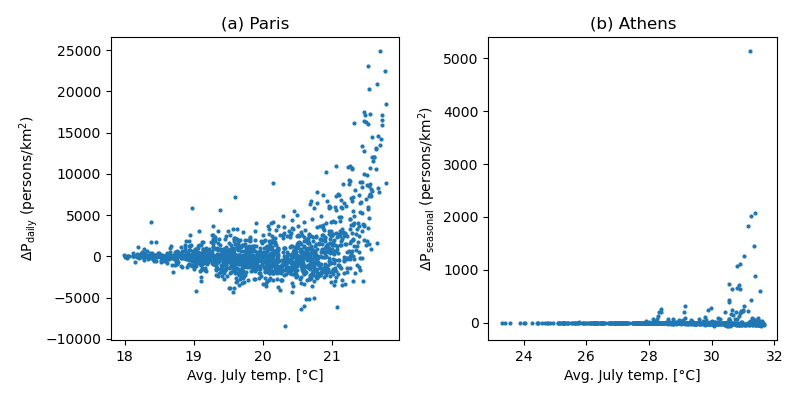

Supplement: S1 Fig — Correlation between ΔPdaily and average temperature in July 2010-2012 in Paris (a). Correlation between ΔPseasonal and average temperature in July 2010-2012 in Athens (b). Each dot shows data of a 1-km grid from the distribution. (PNG) [file pone.0330912.s001.png]

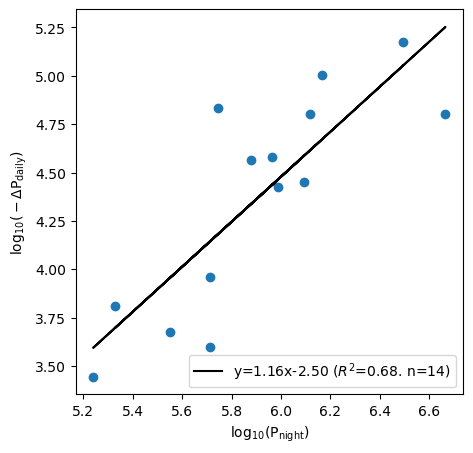

Supplement: S3 Fig — Same figure as Fig 3d but with the 14 cities in quadrants II and III in Fig 2a that has higher nighttime population than daytime population. Noted that a negative sign is in front of ΔPdaily in order to keep the values positive before taking logarithm. (PNG) [file pone.0330912.s003.png]

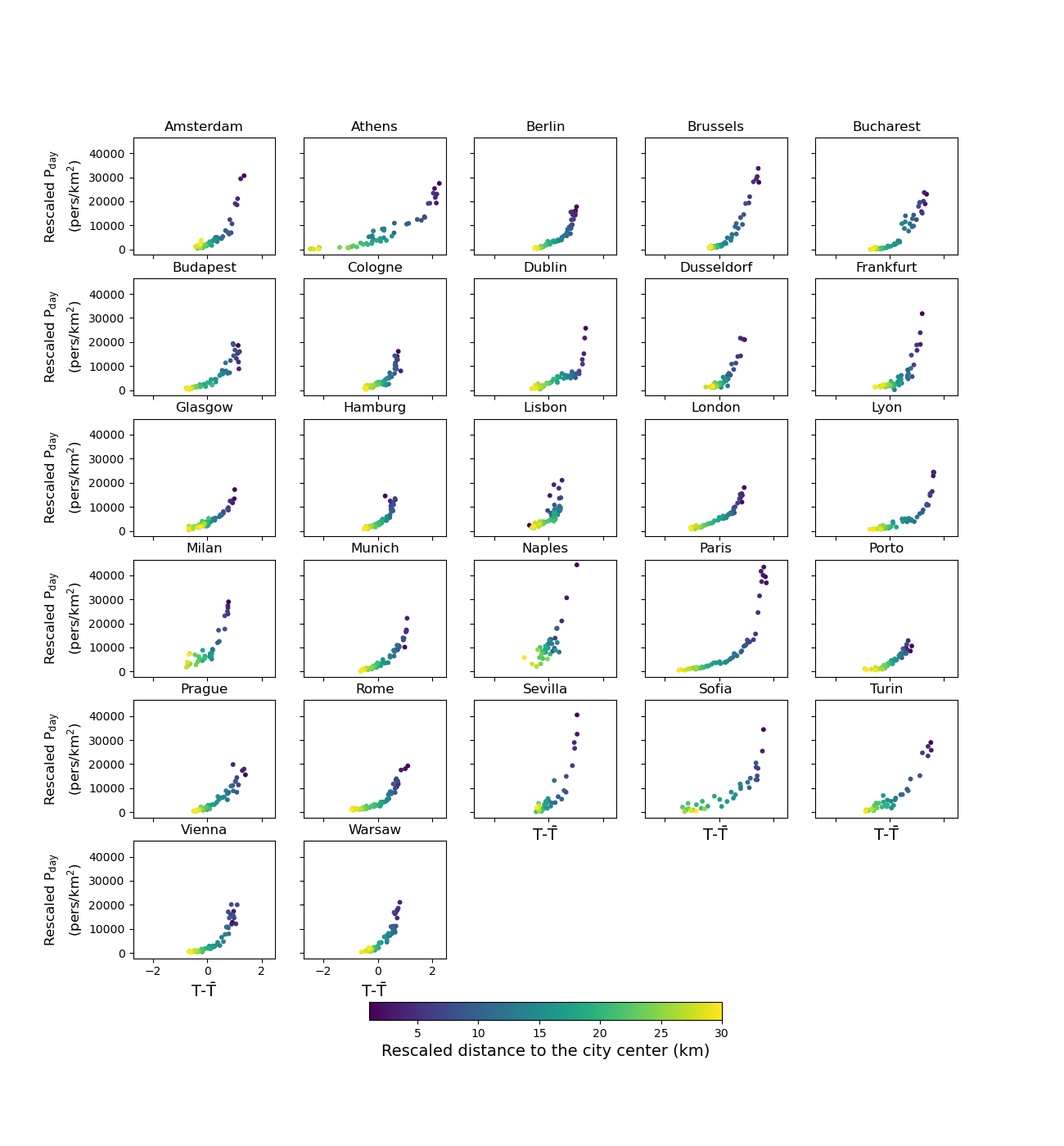

Supplement: S4 Fig — Average rescaled Pday against temperature along the radial profile across the 27 largest European cities (above 1 million) in quadrant I and II in Fig 2a in July. The temperature distribution is from the July average temperature from 2010 to 2012. Temperatures in each city are normalized by subtracting the city’s UrbClim domain’s average temperature. (PNG) [file pone.0330912.s004.png]

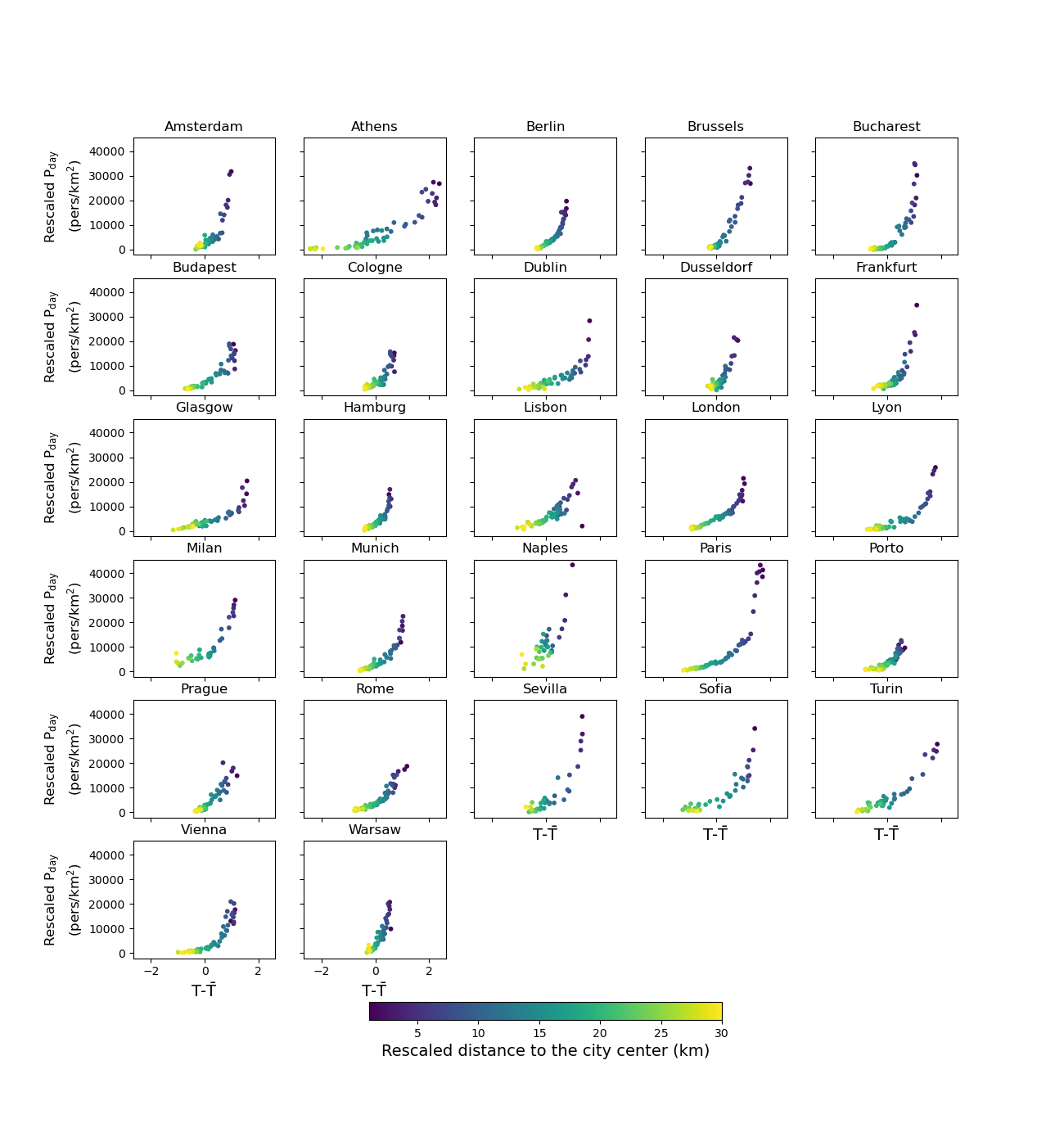

Supplement: S5 Fig — Average rescaled Pday against temperature along the radial profile across the 27 largest European cities (above 1 million) in quadrant I and II in Fig 2a in January. The temperature distribution is from the January average temperature from 2010 to 2012. Temperatures in each city are normalized by subtracting the city’s UrbClim domain’s average temperature. (PNG) [file pone.0330912.s005.png]

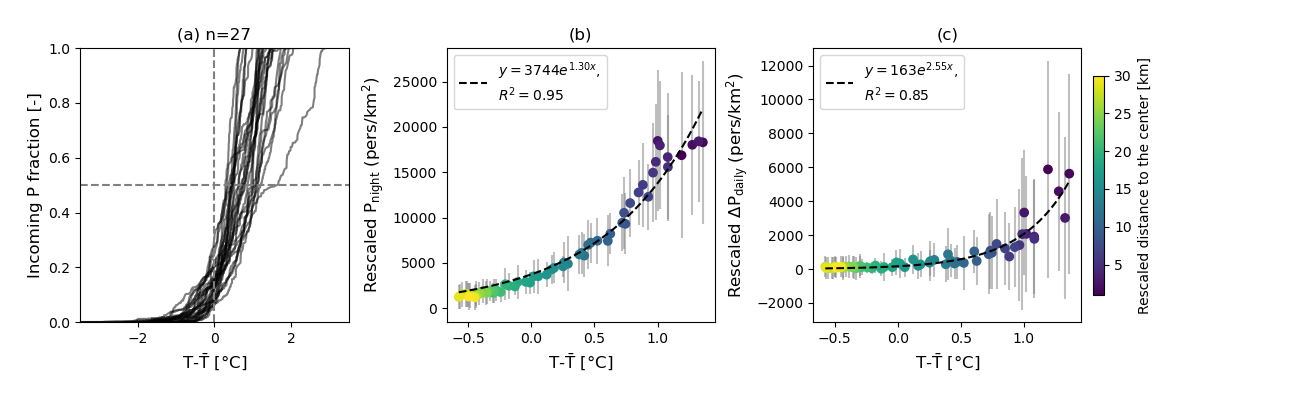

Supplement: S6 Fig — Same figure as Fig 4, but in January. The temperature distribution is from the January average temperature from 2010 to 2012. (PNG) [file pone.0330912.s006.png]

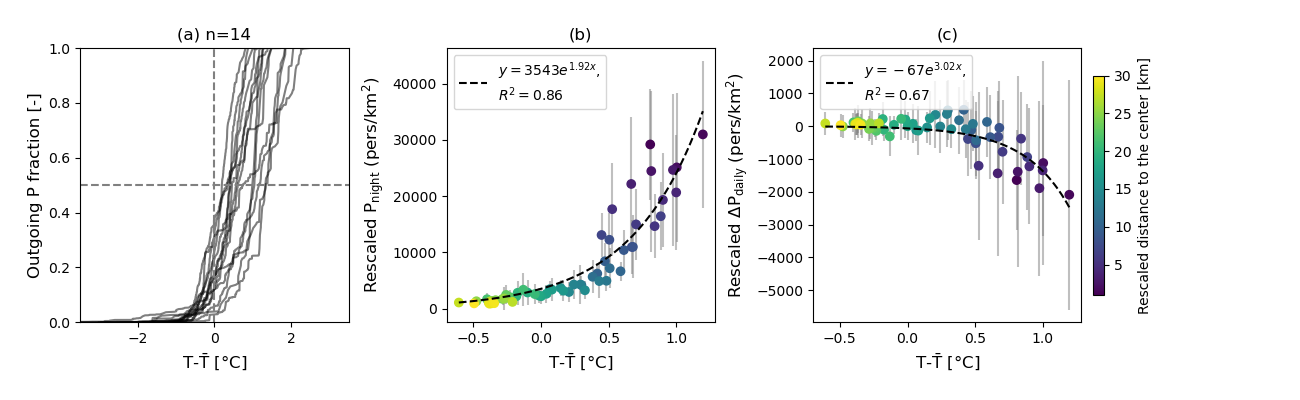

Supplement: S7 Fig — Same figure as Fig 4, but with the 14 cities in quadrants II and III in Fig 2a that show lager nighttime population than daytime population. (PNG) [file pone.0330912.s007.png]

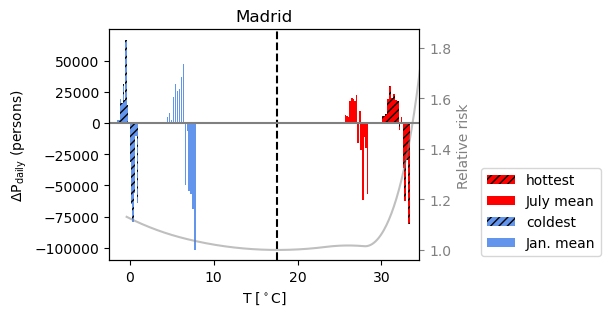

Supplement: S8 Fig — Same as Fig 5a but with Madrid where the dataset [17] shows higher nighttime than daytime population. Daily population mobility changes human heat exposure in Madrid where nighttime population is higher than daytime population. The gray curve is the city’s ERF for the age group 45-65 with the dashed black line marked the MMT. Daily population difference is calculated by daytime minus nighttime population in July. The hatched histograms are plotted with temperature averaged over the warmest/coldest 3% days in July/January from 2010 to 2012. The histograms without hatching are plotted with the monthly average temperature of July/January over the same years. (PNG) [file pone.0330912.s008.png]

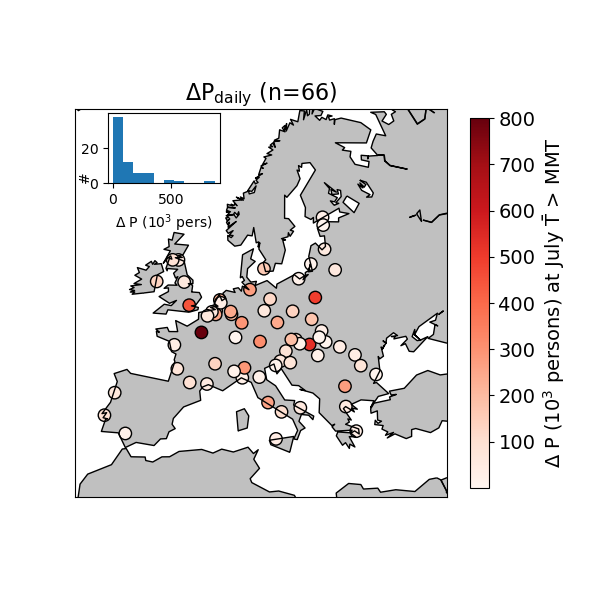

Supplement: S9 Fig — Same figure as Fig 6a but with the average temperature distribution of the hottest 3% days in July 2010-2012. Change in heat exposure is positive in all the cities. (PNG) [file pone.0330912.s009.png]

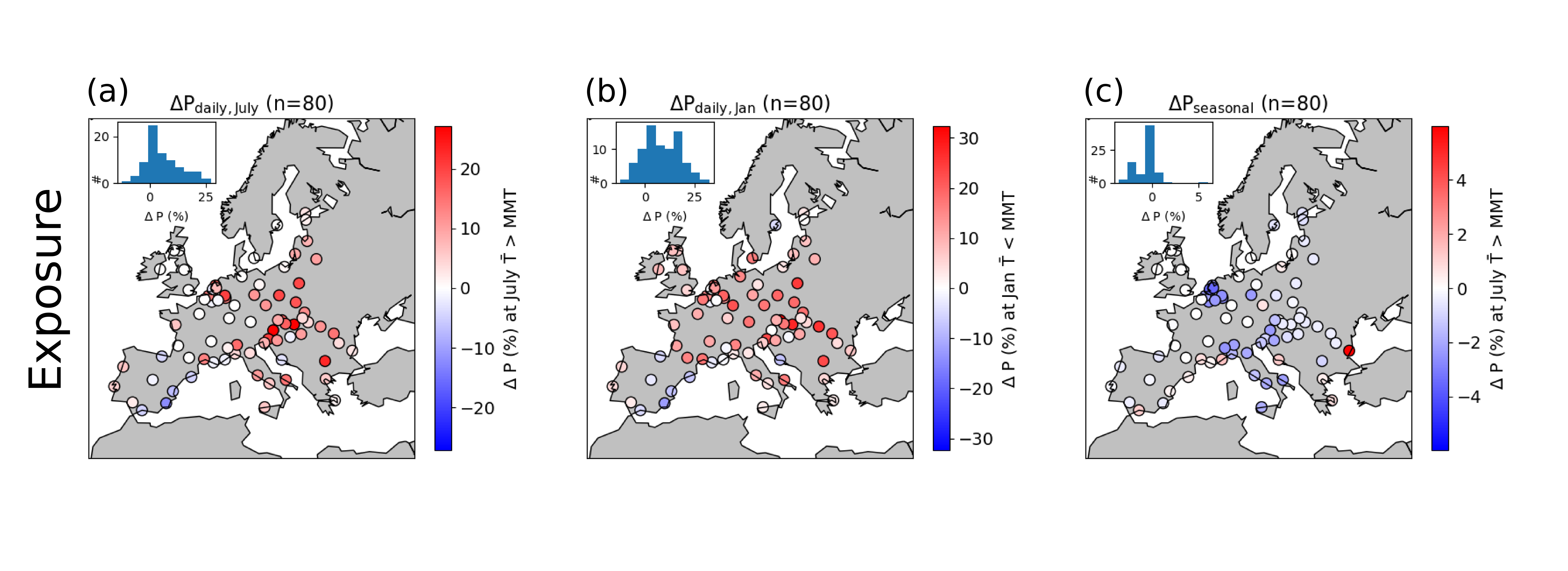

Supplement: S10 Fig — Same as Fig 6a–6c but with adjusted exposure in terms of percentage of July nighttime population (a,c) or January nighttime population (b) for all 80 cities. (PNG) [file pone.0330912.s010.png]

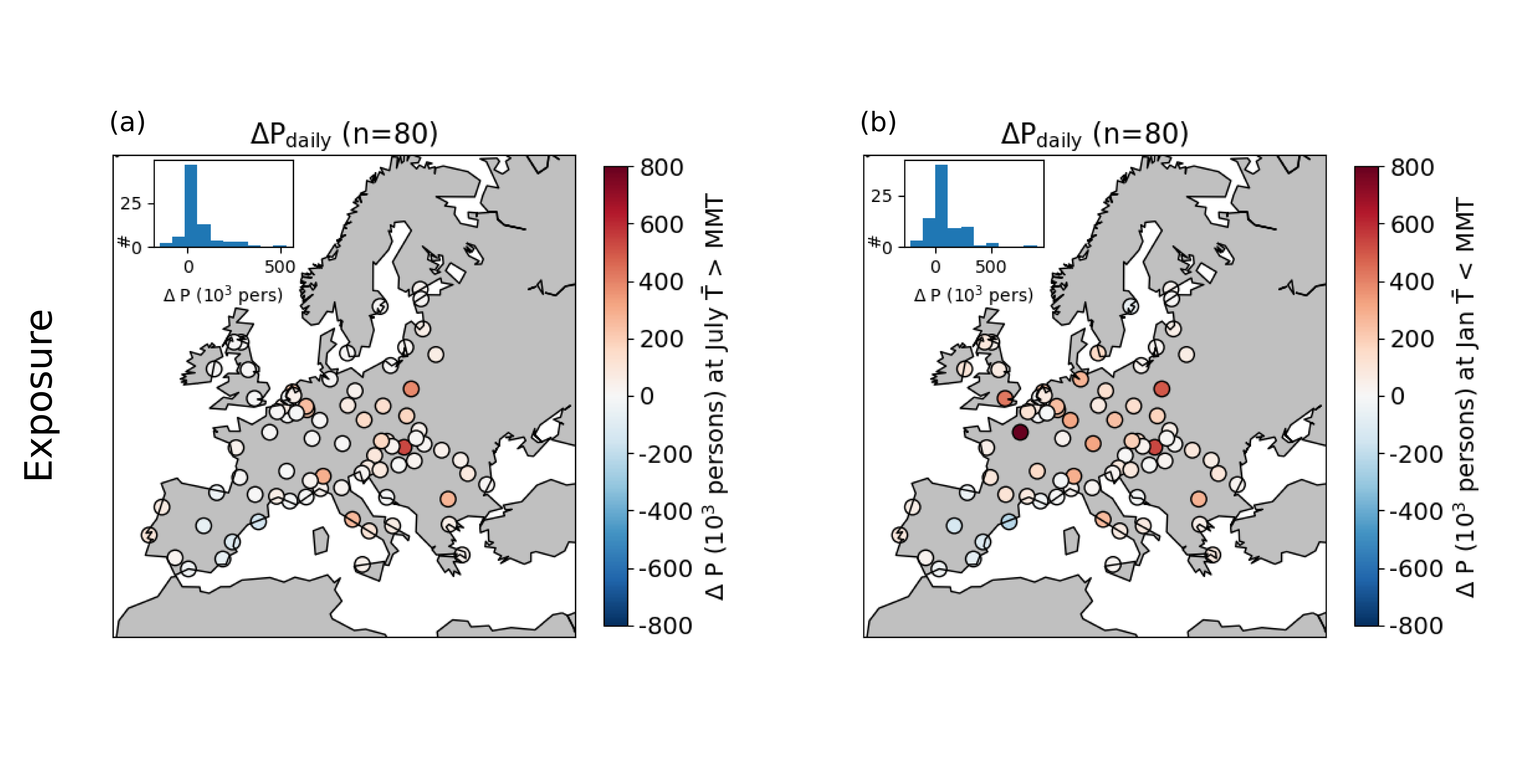

Supplement: S11 Fig — Same as Fig 6a, 6b but with all the 80 cities including the 14 cities where the dataset [17] shows higher nighttime than daytime population. (PNG) [file pone.0330912.s011.png]

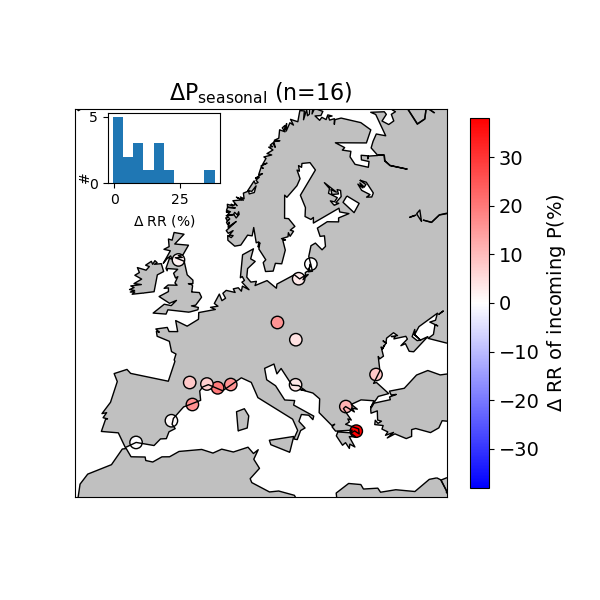

Supplement: S12 Fig — Same figure as Fig 6f but with the average temperature distribution of the hottest 3% days in July 2010-2012. Heat risk increases in all the cities except Malaga where population is estimated to move to the beach on the colder outskirts of the city. The increase in RR is significantly higher than the values shown in Fig 6f where the July monthly average temperature was considered. Noted that for 2 cities (Thessaloniki and Klaipeda) ΔRR is only calculated by taking the maximum RR on the warmer side of the ERF as estimates because the incoming population-weighted temperatures are above the temperature range in the UrbClim temperature-derived ERFs by Huang et al. (2023) [19]. Therefore, the actual ΔRR may be even higher. (PNG) [file pone.0330912.s012.png]
